# A low-cost stand-alone platform for measuring motor behavior across developmental applications

**DOI:** 10.1016/j.isci.2021.102742

**Published:** 2021-06-17

**Authors:** Andrea Cavallo, Nathan C. Foster, Karthikeyan Kalyanasundaram Balasubramanian, Andrea Merello, Giorgio Zini, Marco Crepaldi, Cristina Becchio

**Affiliations:** 1 Cognition, Motion and Neuroscience Laboratory, Center for Human Technologies, Istituto Italiano di Tecnologia, Genova, Italy; 2 Department of Psychology, University of Turin, Torino, Italy; 3 Electronic Design Laboratory, Center for Human Technologies, Istituto Italiano di Tecnologia, Genova, Italy

**Keywords:** Biological sciences, Neuroscience, Behavioral neuroscience, Techniques in neuroscience

## Abstract

Motion tracking provides unique insights into motor, cognitive, and social development by capturing subtle variations into how movements are planned and controlled. Here, we present a low-cost, wearable movement measurement platform, KiD, specifically designed for tracking the movements of infants and children in a variety of natural settings. KiD consists of a small, lightweight sensor containing a nine-axis inertial measurement unit plus an integrated processor for computing rotations. Measurements of three-dimensional acceleration using KiD compare well with those of current state-of-the-art optical motion capture systems. As a proof of concept, we demonstrate successful classification of different types of sinusoidal right arm movements using KiD.

## Highlights

• We developed a wearable platform (KiD) designed for tracking movements of children
• We establish the potential of KiD as stand-alone movement measurement platform
• We demonstrate successful classification of different types of movements using KiD
• Accelerations collected using KiD compare well with those of optical MoCap systems

## Graphical abstract

**Figure:**
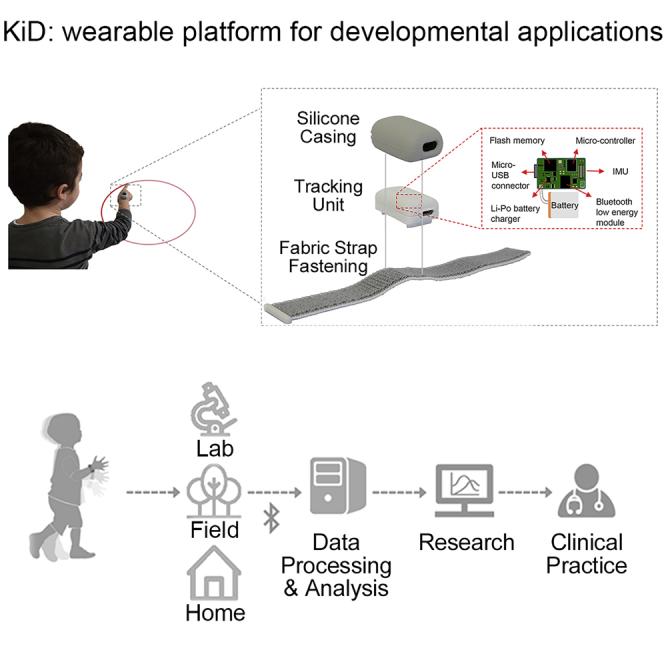

## Introduction

Children's behavior is dynamic, complex, and highly variable, both within and across individuals. This presents challenges for both quantifying it and connecting it with neural development (Adolph, 2016). Since the inception of developmental psychology, researchers have pursued methods to robustly and accurately measure infants' and toddlers' changing behavior. Over the last decade, the increasing availability of high-resolution, high-speed optical cameras has opened the possibility to capture and quantify behavior at an unprecedented level of detail. Optical motion capture systems estimate the 3D position of retroreflective markers placed on the actor's body via triangulation from multiple, static, calibrated infrared 2D cameras, and provide submillimeter accuracy and precision. Combining optical motion capture with machine learning methods of movement analysis has led to breakthroughs in dissecting the sensorimotor and cognitive processes associated with the planning and control of upper limb-reaching movements in both typical and atypical development (e.g., Cavallo et al., 2021; Crippa et al., 2015; Vabalas et al., 2020). However, the requirement for a highly controlled environment, together with the high costs, constrain the routine use of optical (e.g., van der Kruk and Reijne, 2018; van Schaik and Dominici, 2020) motion capture systems in developmental settings.

In recent years, miniaturization of inertial measurement units (IMUs) has paved the way toward an alternative approach to motion capture, inertial motion tracking. IMUs are typically composed of micro-accelerometers, micro-gyroscopes, and micro-magnetometers to measure linear accelerations, angular velocity, and angular orientation within 3D space. Owing to their capability to directly measure body segment movement in any environment with no restrictions on capture volume, as well as being comparatively easier to use and more economical than optical systems (Iosa et al., 2016), IMUs are being increasingly used in health care, ergonomics, sport, entertainment, and industry, with a variety of commercial IMU-based solutions available for stand-alone data collection (e.g., Chambers et al., 2015; Reenalda et al., 2016).

The deployment of IMU platforms in developmental settings presents opportunities for non-intrusive data collection of motor behavior in in-field or in-clinic applications (Campolo et al., 2012; Taffoni et al., 2012). However, the application of IMUs to characterize the motion profiles of children and infants has so far been limited (Busser et al., 1997; Plötz et al., 2012). One reason is the lack of out-of-the-box solutions designed to address the constraining, and often conflicting, requirements of inertial motion tracking in developmental settings. For example, to ensure a natural movement regime, sensors must operate wirelessly and be small and lightweight, which puts a restriction on battery size and longevity. The likelihood of tiredness for young children and infants (and restlessness in older children) requires that procedures for data acquisition and transfer be easy and fast. Moreover, to allow for extra breaks or protocol modifications (often needed in infant research), the experimenter should be able to interface with the device remotely to control the acquisition process.

**Figure 1:**
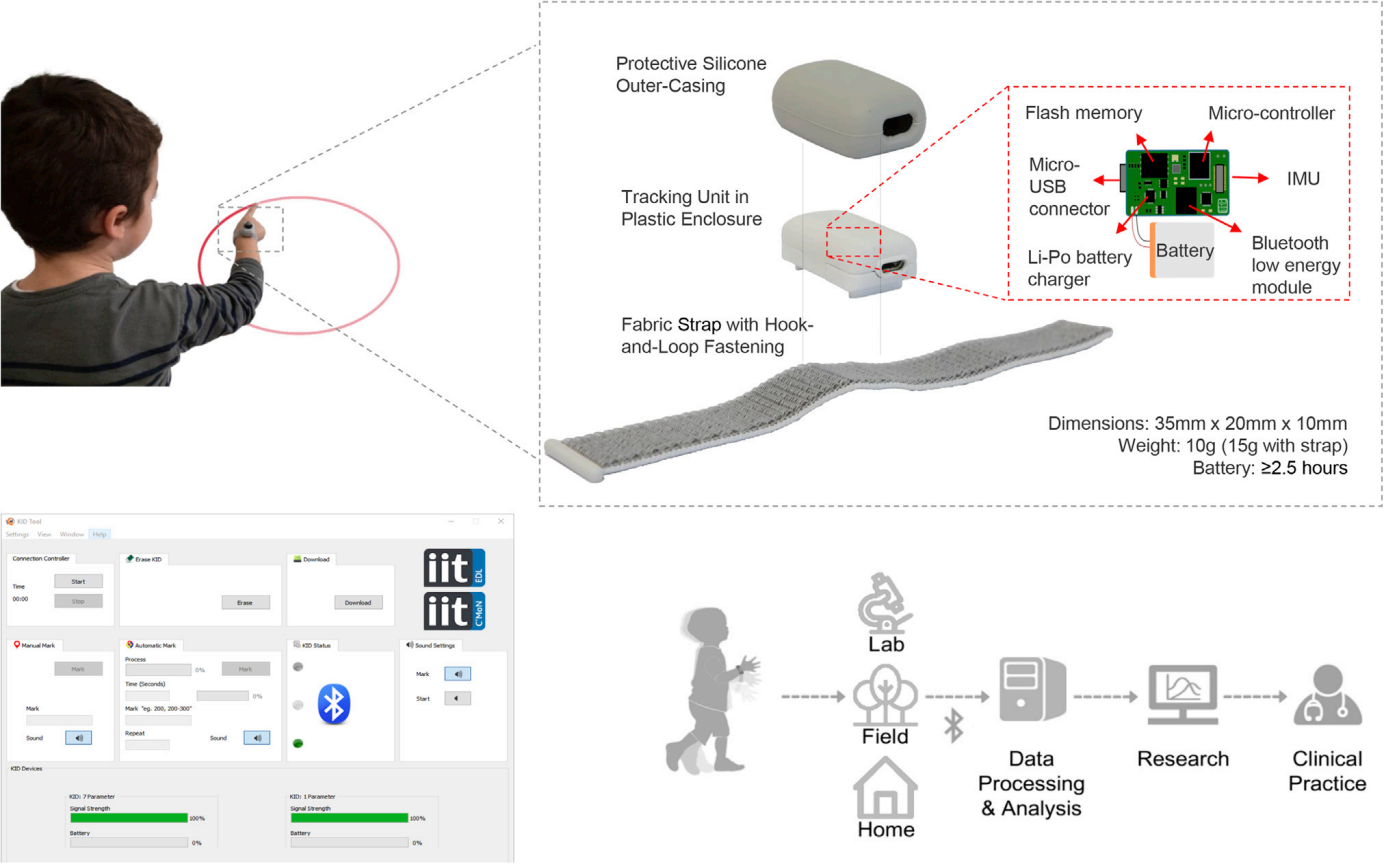
Overview of the KiD platform (Upper panel) The KiD platform being worn by a child while performing an elliptical movement, and exploded view of the KiD tracking unit. (Lower panel) Overview of the KiD user interface (UI), and block diagram of the potential use of KiD as a stand-alone platform for measuring motor behavior across developmental applications.

In the present study, we capitalized on IMU sensors to develop a low-cost, wearable, movement measurement platform, KiD, that addresses these requirements (Figure 1). The key features of KiD are (1) the soft feel, unobtrusive ergonomic design, and small form factor; (2) the ultra-low-power and low-latency radio transmission and high inertial quantities sampling rate; (3) the versatile, easy-to-use, graphical user interface (GUI) designed to allow users to remotely control data acquisition and transfer, including online event labeling; and (4) the basic set of functions, developed in MATLAB, to support offline data analysis without any prior programming experience. Importantly, both the GUI and the MATLAB functions can be run on a standard computer.

With the rationale of establishing the potential of KiD as a stand-alone platform for reliable and fast quantitative motion analysis in real-world settings, we verified the capability of KiD to track and process sinusoidal arm movements in a sample of 20 school-aged children. The value of precisely tracking sinusoidal arm movements to uncover kinematic laws of motion and their systematic violations have been demonstrated in both typical (e.g., Schaal and Sternad, 2001) and atypical populations (e.g., Cook et al., 2013). We evaluated the accuracy of KiD against a “gold-standard” optical motion capture system in reconstructing wrist acceleration profiles of sinusoidal movements performed with the right hand. We also applied classification algorithms to provide a proof of concept that KiD data can accurately differentiate between multiple movement patterns of children.

## Results

### KiD design

KiD platform utilizes a wearable, battery-powered device, incorporating an IMU comprising a three-axis accelerometer, a three-axis gyroscope, and a three-axis compass, which outputs rotation data in quaternion form. The tracking unit, encapsulated in a protective silicone outer casing, can be secured around the wrist or ankle with a hook-and-loop fastener. The compact, deliberately inconspicuous design and the light weight ensure that the device is comfortable and non-distracting to infants' and children's motor performance. The device is micro-controlled and comprises a Li-Po battery charger. An internal flash memory permits the storage of high-fidelity inertial data (sampling frequency up to 200 Hz) to be transferred to external devices both through an on-board Bluetooth low-energy module and a wired micro-USB interface. A set of custom MATLAB functions (MathWorks, Natick, MA) is provided to support the analysis of KiD data (see STAR methods for a detailed description of the KiD platform). A block diagram of the device is shown in Figure 1.

### KiD reliability as a stand-alone platform

**Figure 2:**
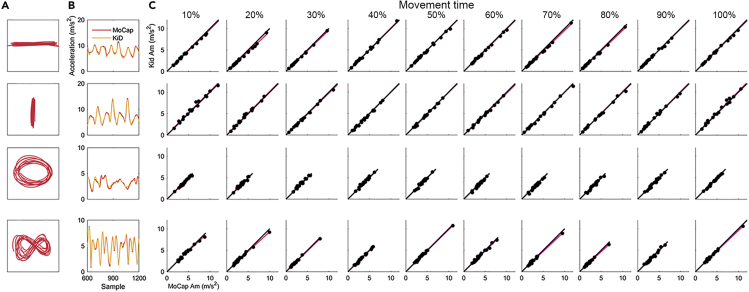
Similarity between the magnitude of motion acceleration measured using KiD and MoCap during horizontal, vertical, elliptical, and figure eight movements (A) Representation of movement performed by an exemplar participant. (B) Magnitude of motion acceleration (Am) measured by KiD and MoCap across samples of an exemplar participant. (C) Scatterplot of the Am measured by MoCap against the Am measured by KiD across individual participants at intervals of 10% of the normalized movement time. Each dot represents the average acceleration for each subject. Black line represents the line of equality between MoCap and KiD acceleration. Red line is a trend line (least squares line) passing through the observed values.

To establish the potential of KiD as a stand-alone platform for monitoring the dynamic motion of human limbs, we compared acceleration profiles measured by KiD with acceleration data captured by an optical motion capture system (Vicon Motion Systems Ltd.; hereafter MoCap). KiD data and MoCap data were collected simultaneously from 20 children (aged 7–11 years) performing four types of sinusoidal right arm movements (Figure 2A; STAR Methods). As shown in Figure 2B, acceleration profiles measured by KiD and MoCap showed a nearly perfect visual overlap across the four types of movement.

To quantify the similarity between the magnitude of motion acceleration (Am) measured using KiD and MoCap, we estimated intraclass correlation coefficients (McGraw and Wong, 1996) for consistency—the extent to which Am measured by KiD and MoCap retain positive correlation, regardless of differences in absolute values—and absolute agreement—the extent to which KiD and MoCap yield similar absolute values of Am separately for each type of movement (see STAR methods for details about data analyses). Across movement types, consistency values ranged from 0.981 to 0.999 (Table S1). Absolute agreement values were also close to 1, indicating near-perfect agreement between the absolute values of Am recorded using KiD and MoCap (Table S1 and Figure 2C). Overall, these results indicate the potential for using KiD to quantitatively assess acceleration profiles of different types of movements.

### Classification of types of movement

**Figure 3:**
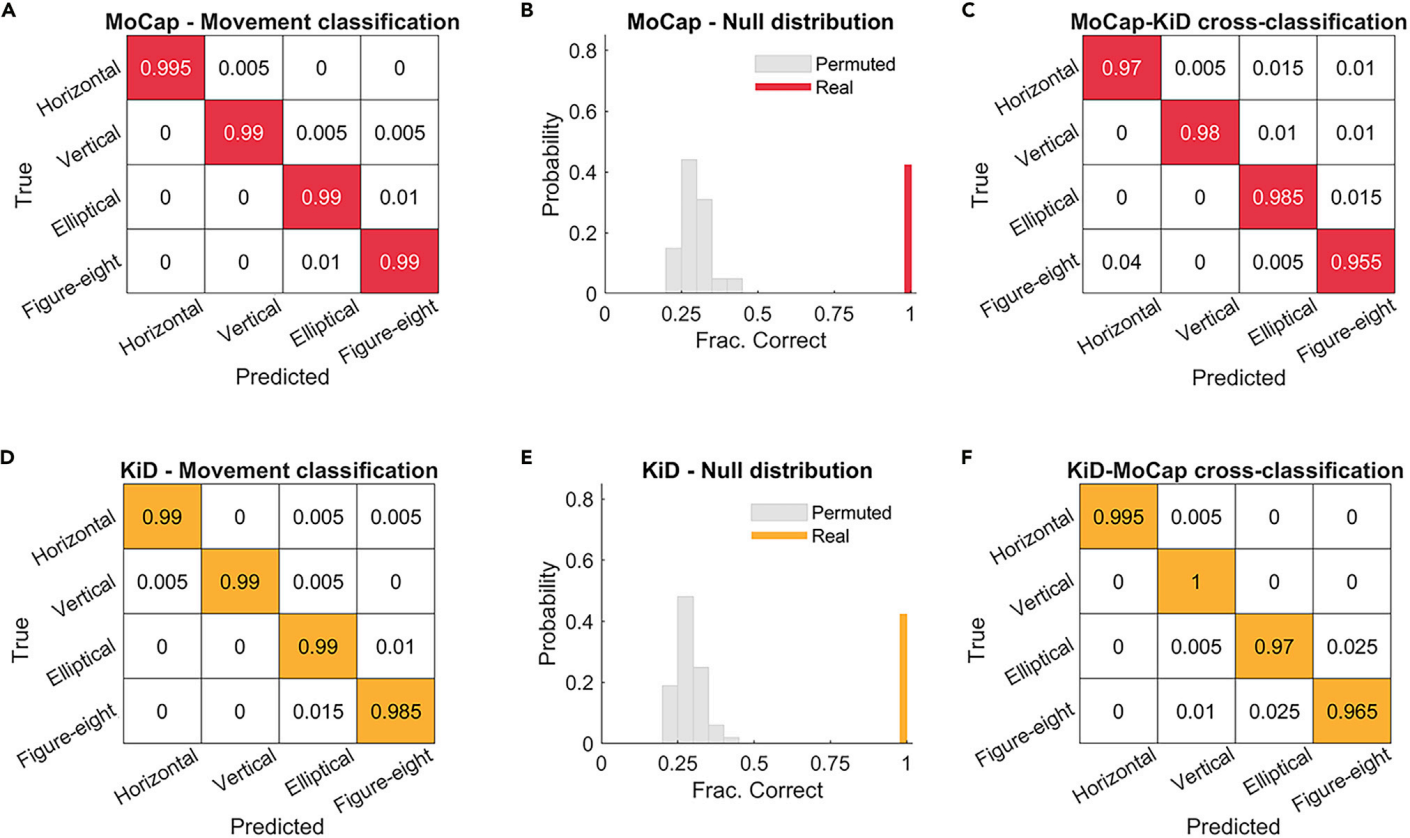
Classification of movement types (A) Confusion matrix of SVM classifier trained and tested on MoCap data. Rows represent the percentage of true movement-type labels. Columns represent the percentage of predicted movement-type labels. (B) Permutation null distribution obtained by classifying MoCap with shuffled movement-type labels. The permutation null distribution is represented by the gray histograms. The red line represents the observed accuracy. (C) Confusion matrix of SVM classifier trained on MoCap data and tested on KiD data. (D) Confusion matrix of SVM classifier trained and tested on KiD data. (E) Permutation null distribution obtained by classifying KiD with shuffled movement-type labels. The permutation null distribution is represented by the gray histograms. The yellow line represents the observed accuracy. (F) Confusion matrix of SVM classifier trained on KiD data and tested on MoCap data.

To verify the capability of our device in identifying specific movement patterns, we applied a machine-learning-based classifier on KiD tri-axis motion acceleration (Ax, Ay, Az) to classify different types of movements (horizontal, vertical, elliptical, and figure eight). For comparison, we trained and tested the same classifier on MoCap tri-axis motion acceleration data (see STAR methods). Classifier performance on MoCap data achieved near-perfect classification accuracy (mean ± SEM = 0.991 ± 0.004; p value after 100 permutations <0.001; Figures 3A and 3B). Classification performance on KiD data was also near perfect (mean ± SEM = 0.989 ± 0.004; p value after 100 permutations <0.001; Figures 3D and 3E).

To substantiate the similarity between acceleration patterns measured using KiD and MoCap, we next trained the classifier to distinguish movement types on MoCap data and tested its classification ability on KiD data (and vice versa, trained the classifier on KiD data and tested it on MoCap data) using a cross-classification approach (Kaplan et al., 2015). The accuracies obtained from the two directions of cross-classification were again near perfect (MoCap-KiD cross-classification accuracy: mean ± SEM = 0.974 ± 0.006, p value after 100 permutations <0.001; KiD-MoCap cross-classification accuracy: mean ± SEM = 0.982 ± 0.006, p value after 100 permutations <0.001; Figures 3C and 3F).

## Discussion

IMU is a powerful technology with a wide range of applications in rehabilitation, ergonomics, industry, sports training, and entertainment. We proposed a wearable IMU-based platform, KiD, designed for developmental applications. To establish the capability of KiD as a stand-alone platform for tracking acceleration profiles, we compared the performance of KiD against that of an optical marker-based motion capture system (MoCap) during performance of sinusoidal arm movements. Experimental results revealed near-perfect agreement between the magnitude of motion acceleration measured using KiD and MoCap. The capability of tri-axis acceleration KiD data to identify movement types was also near perfect, as it was for MoCap. Moreover, the movement-specific acceleration patterns measured through KiD and MoCap largely overlapped, as demonstrated by successful cross-classification from KiD to MoCap (and vice versa). Combined, these analyses demonstrate that KiD can be reliably used for easy and fast quantitative assessments of motion profiles in real-world settings.

In the future, we plan to extend our approach to examine the capability of KiD in identifying more subtle patterns within and between neurotypical children and children diagnosed with neurodevelopmental disorders such as autism. Kinematic studies have already shown that individuals with autism spectrum disorders conducting sinusoidal arm movements move differently—specifically, with greater jerk, velocity and acceleration—relative to typical individuals (Cook et al., 2013). Moreover, autism-related kinematic differences have been documented in the development of prospective control of grasping (Cavallo et al., 2018). When combined with machine learning approaches, these differences may be able to serve as objective markers of autism (e.g., Cavallo et al., 2021; Crippa et al., 2015; Vabalas et al., 2020) and ultimately improve the standard of diagnosis and treatment (Torres and Donnellan, 2015). However, despite the increasing recognition that motion tracking provides unique insights into typical and atypical motor, cognitive, and social development, the use of motion capture in the clinical landscape remains specialist. In the United Kingdom and Ireland, for example, in 2020 there were only 13 gait laboratories accredited by the Clinical Movement Analysis Society (CMAS; https://cmasuki.org/laboratories/). Our platform is dramatically lower in cost than optical motion capture and does not require specialized equipment or training. By making motion tracking more accessible, easy to use, and cost effective, while maintaining measurement accuracy, KiD holds a great deal of promise for the greater use of motion measurements (including the possibility for remote data collection) in the assessment of the treatment of developmental disorders (Bisi et al., 2017; Lander et al., 2020).

### Limitations of the study

We evaluated the capability of the KiD platform in identifying whole-arm movements performed by typically developing children. For a robust deployment of KiD in real-world and clinical settings, future studies should establish the capability of KiD in identifying movement patterns over a wider range of motor activities and populations. Also, for in-clinic applications, future studies will have to demonstrate the feasibility of using KiD, in conjunction with machine learning approaches, for developing protocols and deriving metrics that can detect clinically meaningful changes in function (e.g., disease-specific changes in upper- or lower-limb kinematics) and inform the efficacy of intervention (e.g., by evaluating function before and after intervention).

## STAR★Methods

### Key resources table

**Table:** 

REAGENT or RESOURCE	SOURCE	IDENTIFIER
**Deposited Data**		

Data supporting main findings	This paper	Mendeley Data: https://doi.org/10.17632/dzdcw2rw5k.1

**Software and Algorithms**		

Vicon Nexus	Vicon Motion Systems Ltd UK	https://www.vicon.com/products/software/nexus; RRID:SCR_015001
MATLAB	MathWorks Inc	https://it.mathworks.com/products/matlab.html; RRID:SCR_001622
Python	Python software foundation	http://www.python.org/; RRID:SCR_008394

### Resource availability

#### Lead contact

Further information and requests for resources should be directed and will be fulfilled by the Lead contact, Cristina Becchio (cristina.becchio@iit.it).

#### Materials availability

This study did not generate new unique reagents or materials.

#### Data and code availability

The data supporting the main findings of this study are available for download from Mendeley data: https://data.mendeley.com/datasets/dzdcw2rw5k/1. The code supporting the main findings of this study is based on public available tools listed in the Key Resource Table. KiD User Interface and custom MatLab functions developed to support collection, processing and analyses of KiD data will be made available by the Lead contact upon reasonable request.

### Experimental model and subject details

We report results about movement traces collected from 20 children (5 females) aged 7-11 years (mean ± SD = 9.5 ± 1.2 years.months). All participants were right-handed, had normal or corrected to normal vision, and no history of developmental or neurological disorders. The research protocol was approved by the local ethics committee (Comitato Etico Regionale Liguria) and was in accordance with the principles of the revised Helsinki Declaration (World Medical Association, 2013). Parents provided written informed consent after receiving a detailed description of the study.

### Method details

#### Design of the wearable device

The KiD platform utilises a wearable, battery-powered wristband-type tracker that records and stores inertial data on an internal memory. All the internal devices composing the KiD (electronics and battery) are placed inside a plastic enclosure (35 x 20 x 10 mm), which is further encapsulated within a protective silicone outer casing (40 x 25 x 15 = 15000 mm^3^) for a total weight of 15 g. The KiD can thus be secured around the wrist or ankle with a hook-and-loop fastener. The shape, dimensions, weight and functionalities of the KiD have been specifically conceptualized and designed for applications with infants and children in both laboratory and ecological settings. In particular, the external silicone case is designed with a double purpose: i) ensure that KiD is comfortable and non-distracting to children by covering any light emitted by the device; ii) enable immediate technical intervention in case of electronic failure during data collection.

Internally, the KiD platform is equipped with a commercial micro-controller unit (STM32L476, ST Microelectronics), an Inertial Measurement Unit (IMU, MPU-9250, Invensense) with a three-axis accelerometer, a three-axis gyroscope, a three-axis compass and an integrated processor that computes rotation data in quaternion form, a Bluetooth low energy module (BGM123, Bluegiga), a LiPo battery charger/voltage regulator (BQ24230, Texas Instruments) and an on-board Quad Serial Peripheral Interface (Quad-SPI) flash memory (S25FL256SAGNFI001, Spansion/Cypress). The micro-controller unit, which runs FreeRTOS (https://www.freertos.org), is in charge of acquiring data from the IMU, handling Bluetooth radio and flash memory and interfacing to the LiPo charger to monitor battery drain. The micro-controller is powered at 3.3V from the LiPo charger/voltage regulator while its main clock is 80MHz for a Cortex-M4 ARM core. The measuring range provided by the IMU is configurable from ± 2G (± 19.61m/s^2^; optimal for measuring, with high resolution, fine motor movements) to ± 8G (± 78.45m/s^2^; optimal for measuring quick movements), while the ranges of gyroscope and compass are ± 2000°/s and ± 4912μT, respectively. Bluetooth low energy (BLE) module implements physical radio, Generic Attribute Profile (GATT) services and enables on-board passive antennas without requiring external components. BLE module keeps the KiD battery size small while guaranteeing a capacity adequate for a typical recording session with children and infants (> 2.5 consecutive hours of recording). All the Bluetooth chips have a unique MAC address to access them. This makes each single KiD simply distinguishable in experimental settings that require the contemporary use of multiple platforms (e.g. data collection from more than one limb, or simultaneous collection from multiple children). The LiPo charger module recharges the LiPo battery using the energy provided by a micro USB port and generates DC regulated voltage for all the on-board modules. The flash memory of the KiD (256 Mb) saves the IMU data and can also store markers sent by the user through the BLE module. The data stored on the internal memory can be transferred to external devices by means of both the Bluetooth connection and a micro USB connection embedded on the device. A diagram of the KiD device is shown in Figure 1.

The KiD platform is complemented by a User Interface (UI) that supports the direct ad-hoc data transfer between the KiD and a personal computer, both via Bluetooth or micro USB. Moreover, the UI allows users to monitor the device status and the connectivity strength, and to send markers during data acquisition, with a latency lower than 7.5 ms. The main commands that can be sent to the KiD via the UI are:

**START**: To begin the acquisition process (store the IMU values to the memory).

**STOP**: To pause the acquisition process.

**MARK**: To store markers on to the memory during acquisition process.

**DOWNLOAD:** to download and save the data on an external device,

**ERASE**: To erase the KiD memory.

A set of custom MATLAB functions (MathWorks, Natick, MA) has also been developed to support analyses of KiD data. They allow the user to: i) upload KiD data to the MATLAB environment; ii) identify the markers sent during data acquisition and use the markers to segment the data; iii) apply a low-pass Butterworth filter to the data, with the full possibility to personalize the cut-off frequency; iv) convert inertial data from KiD coordinates into world coordinates; v) synchronize KiD and motion capture (MoCap) data by means of cross-correlation analyses (in case of KiD and MoCap simultaneous recordings).

Both the UI and the MATLAB functions can be run on a standard PC. The use of MATLAB functions needs minimal programming experience. In addition to functions, a MATLAB script has been written in order to allow users to run the functions in a semi-automatic way, where only the filename containing raw KiD data needs to be specified by the user.

#### Dataset of children’s hand motions

Participants were instructed to perform four types of right-hand movements: horizontal, vertical, elliptical and figure-eight.

*Horizontal* movements: participants were instructed to conduct simple horizontal sinusoidal (left and right) right arm movements of about 40 cm amplitude.

*Vertical* movements: participants were instructed to conduct vertical sinusoidal (up and down) right arm movements of about 50 cm amplitude.

*Elliptical* movements: participants were instructed to perform elliptical sinusoidal right arm movements. A drawing of an ellipse (of major axis 22.5 cm and minor axis 15.1 cm) was provided on a stand in front of the child as template. Movements were accompanied by an auditory tone which encouraged participants to move at approximately the same rate.

*Figure-eight* movements: participants were instructed to perform figure-eight sinusoidal right arm movements. As for elliptical movements, a drawing of a figure-eight curve was provided on a stand in front of participants and movements were accompanied by an auditory tone.

For all movement types, the starting position was upright, with the right arm stretched out at approximately 90° with respect to the medio-lateral axis of the trunk. After a training phase, participants completed ten cycles of each type of movement (for horizontal/vertical movement, moving from left/up to right/down and returning made up a cycle; for elliptical and figure-eight movements, completing the shape made up a cycle). The order of movements was pseudorandomized across participants.

KiD data were collected simultaneously with MoCap data. To do so, participants wore KiD on the right wrist and, affixed to KiD, a retroreflective-marker (6.5 mm in diameter). An optical motion capture system equipped with eight infrared cameras (Vicon, Vicon Motion Systems Ltd., UK) was used. Both KiD and MoCap data were collected at a sampling rate of 200 Hz (compass at 15 Hz).

### Quantification and statistical analysis

#### Data processing

After data collection, both KiD and MoCap data were run through a low-pass Butterworth filter with a 6 Hz cutoff. The accelerometer data of the KiD where converted from device coordinates into world coordinates by means of the rotation matrix obtained from the quaternion. In this way, independently from the orientation of the KiD, left-right movements of the arm resulted in accelerations on the x-axis, up-down movements resulted in accelerations on the z-axis, and backward-forward movements resulted in accelerations on the y-axis. The same reference system was used for acceleration data computed as the second derivative of position using MoCap. After rotation, x-, y-, z-acceleration (Ax, Ay, Az) and acceleration magnitude (Am) were computed for both KiD and MoCap data.

#### Data analysis

We synchronized KiD and MoCap by computing cross-correlation between vectors of Am. The Am vector of MoCap was kept fixed and the Am vector of KiD was shifted (lagged) until the best correlation between the two vectors was obtained. The resulting sample-lag was then used to cut the initial Am samples of the KiD. Finally, to obtain vectors of the same length, the final Am samples of the longest vector were cut. Ax, Ay and Az vectors were cut and synchronized using the same sample-lag obtained from Am. After synchronization, Ax, Ay, Az and Am of both KiD and MoCap were segmented in order to obtain, for each movement series, 10 vectors, each of them representing accelerations of a movement cycle. The final dataset then consisted of a total of 800 movements (20 participants x 4 movement types x 10 cycles). For each cycle, accelerations were expressed with respect to normalized (%) cycle duration and resampled at intervals (time-bins) of 10% of the normalized cycle duration.

#### Agreement between KiD and MoCap

In order to evaluate agreement between KiD and MoCap, we computed Intra-class Correlation Coefficients (ICCs, McGraw and Wong, 1996) of Am. We averaged for each time-bin Am across the ten movement cycles of each participant, separately for each movement type. Next, we used ICCs to compute consistency and absolute agreement.

Consistency was defined as:

$$Consistency=\;\frac{MS_{R}-\;MS_{E}}{MS_{R}}$$

Absolute agreement was defined as:

$$Absolute\;agreement=\;\frac{MS_{R}-\;MS_{E}}{MS_{R}+\;\frac{MS_{C}-\;MS_{E}}{n}}$$

Where *MS*_*R*_ is the mean square for rows, *MS*_*E*_ is the mean square error, *MS*_*C*_ is the mean square for columns and *n* is the number of observations.

We assessed the significance of consistency and absolute agreement under the null hypothesis of ICC = 0 using *F* statistic. Bonferroni adjusted p-values were computed to control for type-1 error deriving from multiple comparisons.

#### Classification of type of movement

We computed classification of type of movement (horizontal, vertical, elliptical, figure-eight) from acceleration data (Ax, Ay, Az) based on a linear Support Vector Machine (SVM), separately for KiD and MoCap data. Models were trained, validated, and tested by means of a 10-fold nested cross-validation (CV) procedure. At each iteration of the 10-fold procedure, we split data in order to obtain a test set of 80 movement cycles (i.e., 4 cycles for each participant, 1 for each type of movement). Hyper-parameters were tuned on the reduced training set by recursively selecting 80 cycles for the validation set. The model was then tested on the remaining fold.

To further assess the similarity between acceleration patterns obtained using KiD and MoCap, we tested the ability of an SVM classifier trained on MoCap acceleration data to classify movement type using KiD acceleration data (and *vice versa*, train on KiD and test on MoCap; i.e. cross-classification) (Kaplan et al., 2015).

In all analyses, we assessed model performance using classification accuracy. We assessed the significance of classification accuracy against chance with permutation statistics. The chance-level null-hypothesis distribution of these statistics was created by computing classification accuracy after randomly permuting the task labels associated to movement repetitions (100 random permutations).
