# Supplementary material for: A low-cost stand-alone platform for measuring motor behavior across developmental applications
Source: iScience. 2021 Jun 17;24(7):102742. doi: 10.1016/j.isci.2021.102742 (PMC8258968; doi:10.1016/j.isci.2021.102742)
Supplement: Document S1. Table S1 [file mmc1.pdf]

iScience, Volume *24*

Supplemental information

A low-cost stand-alone platform for measuring motor behavior across developmental applications

Andrea Cavallo, Nathan C. Foster, Karthikeyan Kalyanasundaram Balasubramanian, Andrea Merello, Giorgio Zini, Marco Crepaldi, and Cristina Becchio

Table S1. *ICC results: consistency and absolute agreement between KiD and MoCap acceleration magnitude for each type of movement. Related to Figure 2.*

		Time-bin									
ICC Consistency		***10%***	***20%***	***30%***	***40%***	***50%***	***60%***	***70%***	***80%***	***90%***	***100%***
Horizontal	***ICC***	.999	.997	.999	.999	.998	.999	.998	.998	.998	.999
	p	<.001	<.001	<.001	<.001	<.001	<.001	<.001	<.001	<.001	<.001
Vertical	***ICC***	.999	.998	.999	.998	.998	.999	.998	.998	.998	.999
	p	<.001	<.001	<.001	<.001	<.001	<.001	<.001	<.001	<.001	<.001
Elliptical	***ICC***	.994	.981	.989	.991	.988	.988	.989	.987	.992	.997
	p	<.001	<.001	<.001	<.001	<.001	<.001	<.001	<.001	<.001	<.001
Figure-eight	***ICC***	.994	.994	.998	.996	.998	.995	.996	.995	.992	.997
	p	<.001	<.001	<.001	<.001	<.001	<.001	<.001	<.001	<.001	<.001
ICC Agreement											
Horizontal	***ICC***	.998	.994	.998	.998	.998	.998	.997	.998	.998	.999
	p	<.001	<.001	<.001	<.001	<.001	<.001	<.001	<.001	<.001	<.001
Vertical	***ICC***	.999	.998	.999	.998	.998	.998	.998	.998	.999	.999
	p	<.001	<.001	<.001	<.001	<.001	<.001	<.001	<.001	<.001	<.001
Elliptical	***ICC***	.991	.981	.985	.989	.988	.981	.980	.984	.990	.993
	p	<.001	<.001	<.001	<.001	<.001	<.001	<.02	<.001	<.001	<.01
Figure-eight	***ICC***	.994	.994	.998	.995	.998	.994	.995	.994	.991	.997
	p	<.001	<.001	<.001	<.001	<.001	<.001	<.001	<.001	<.001	<.001
